# Effects of the floral phytochemical eugenol on parasite evolution and bumble bee infection and preference

**DOI:** 10.1038/s41598-018-20369-2

**Published:** 2018-02-01

**Authors:** Evan C. Palmer-Young, Austin C. Calhoun, Anastasiya Mirzayeva, Ben M. Sadd

**Affiliations:** 1 Organismic & Evolutionary Biology Graduate Program, University of Massachusetts at Amherst, Amherst, Massachusetts 01003 United States; 2 School of Biological Sciences, Illinois State University, Normal, Illinois 61790 United States; 3 Department of Biology, University of Massachusetts at Amherst, Amherst, Massachusetts 01003 United States

## Abstract

Ecological and evolutionary pressures on hosts and parasites jointly determine infection success. In pollinators, parasite exposure to floral phytochemicals may influence between-host transmission and within-host replication. In the bumble bee parasite *Crithidia bombi*, strains vary in phytochemical resistance, and resistance increases under *in vitro* selection, implying that resistance/infectivity trade-offs could maintain intraspecific variation in resistance. We assessed costs and benefits of *in vitro* selection for resistance to the floral phytochemical eugenol on *C. bombi* infection in *Bombus impatiens* fed eugenol-rich and eugenol-free diets. We also assessed infection-induced changes in host preferences for eugenol. *In vitro*, eugenol-exposed cells initially increased in size, but normalized during adaptation. Selection for eugenol resistance resulted in considerable (55%) but non-significant reductions in infection intensity; bee colony and body size were the strongest predictors of infection. Dietary eugenol did not alter infection, and infected bees preferred eugenol-free over eugenol-containing solutions. Although direct effects of eugenol exposure could influence between-host transmission at flowers, dietary eugenol did not ameliorate infection in bees. Limited within-host benefits of resistance, and possible trade-offs between resistance and infectivity, may relax selection for eugenol resistance and promote inter-strain variation in resistance. However, infection-induced dietary shifts could influence pollinator-mediated selection on floral traits.

## Introduction

Antimicrobial phytochemicals may influence the disease ecology of phytophagous animals, including insects^1–3^. Plants produce an enormous variety of these compounds, which are thought to have evolved for plant defense against abiotic and biotic stressors, including infection^4,5^. The same compounds, derived from plants, can reduce parasite and pathogen infection in animals^1–3,6^. In these plant-animal-parasite systems, phytochemicals may add a further evolutionary pressure to the between- and within-host selection mosaic that acts on parasites and pathogens^7^.

The presence of host-produced, host-ingested, and environmental chemical inhibitors creates strong selection for parasite resistance to the effects of these compounds. Evolved resistance can structure host-pathogen interactions both in the short term, by determining which niches a parasite may occupy, and over evolutionary time, as parasites specialize on co-evolving hosts^8^. Aside from being of clinical and agricultural concern, chronic selection for resistance of parasites and pathogens to specific chemicals may alter natural communities of plants, insects, and pathogens.

Self-medication behaviors, defined as infection-induced alterations in preference and behavior that compromise fitness of healthy hosts but improve fitness in the presence of infection, have been suggested in several insect-parasite systems (reviewed in^1,2^). Most cases involve changes in dietary preferences that result from, and may mitigate, infection^1^. For example, arctiid moth larvae preferred poison hemlock plants and artificial diets high in pyrrolizidine alkaloids that improved survival when infected with parasitoid flies^9,10^; and ants exhibited preferences for antimicrobial hydrogen peroxide when infected with fungi^11^. Pollinators and herbivores may ameliorate infection through phytochemical ingestion, and in some cases hosts appear to prefer these substances when infected. For example, protozoan infection altered monarch butterfly oviposition preferences to favor plants high in cardenolides, which protected developing larvae from infection^6^. In honey bees, *Nosema* inoculation resulted in attraction to sunflower honey, which reduced infection when consumed by infected worker bees^12^; and chalkbrood infection altered collection of antimicrobial resins, which reduced infection when experimentally added to hives^13^.

Bumble bees (*Bombus spp*.) and their trypanosomatid parasite *Crithidia bombi* (Trypanosomatidae) have emerged as a model system for host-parasite evolution and ecology, including the effects of phytochemicals on infection outcomes. Several phytochemicals experimentally reduced infection of *B. impatiens* and *B. terrestris*^14–17^, although anti-parasitic effects varied across experiments that used different *Crithidia* lineages, bee colonies, and nutritional and rearing conditions^18–20^, consistent with established variation in infectivity and virulence due to diet and host-parasite genotype-genotype interactions^21–25^. In two studies, *Crithidia* infection resulted in preference of free-flying bees for high-phytochemical natural and artificial flowers that contained potentially antiparasitic compounds^15,26^, although studies with caged individuals showed no infection-induced changes in preference^20^.

Evolution of parasites may also alter the outcome of infection under different environments. Indeed, under *in vitro* conditions, *C. bombi* strains vary in their resistance to certain phytochemicals. In other trypanosomatids, experimental evolution of drug resistance has shown costs, benefits, and no effects for *in vivo* infectivity^27^. *In vitro* selection for growth with the phytochemicals thymol, eugenol, and a thymol-eugenol blend rapidly increased resistance of *C. bombi* to these compounds, with no apparent costs for *in vitro* growth^28^. However, selection for rapid growth *in vitro* in the absence of phytochemicals came at a cost of reduced infectivity in live bees^29^. Like self-medication behavior, the costs and benefits of evolved resistance may be context-dependent, conferring increased fitness under high-phytochemical conditions but reduced fitness under low-phytochemical conditions.

We used the widespread floral volatile eugenol, which has been found in flowers of over 100 plant species^30^, and experimentally evolved eugenol-resistant *C. bombi* cell lines (generated in prior experiments^28^) to explore the potential role of this phytochemical in the ecology and evolution of *Bombus-Crithidia* host-parasite interactions. Specifically, we analyzed effects of chronic eugenol exposure on *C. bombi* cell morphology during the prior experiment’s *in vitro* adaptation period, and conducted an experiment that tested the effects of dietary eugenol and eugenol selection regime on infection. In addition, we investigated how parasite exposure and infection influences dietary preference for eugenol in live bees. The Infection Intensity Experiment tested how eugenol-containing sugar-water and prior parasite selection for eugenol resistance affected infection intensity and sugar-water consumption under no-choice conditions. We expected that dietary eugenol would reduce overall infection, but that eugenol-containing diets would result in relatively greater infection by eugenol-selected parasites. In contrast, eugenol-free conditions would result in relatively greater infection by the unselected control parasites, due to costs associated with the evolution of phytochemical resistance. The Preference Experiment tested how infection with *C. bombi* affected preference for 50 ppm eugenol-containing vs. 0 ppm control solutions of sugar water. We expected that infection would shift the preference of bees towards increased consumption of putatively antiparasitic eugenol-containing solutions.

## Methods

### Parasite cultures and experimental evolution

Both experiments used *C. bombi* cell lines that originated from wild bumble bees (*Bombus impatiens*) collected near Normal, IL, United States. The cell lines used in the parasite Morphology and Infection Intensity Experiments originated from *C. bombi* collected in 2013 (strain ‘IL13.2’, collected by BMS). Selected lines of this strain, generated during a previous experiment that measured responses to eugenol-mediated selection^28^, were used in these experiments. Briefly, the ancestral strain IL13.2 was divided into 10 independently propagated cell lines. Five of these cell lines were grown in 50 ppm eugenol (Selection regime = Eugenol), the remaining lines were grown without eugenol (Selection regime = Control). Lines were propagated for 6 weeks (approximately 100 parasite generations), during which time the eugenol-selected lines showed a ~10% increase in eugenol resistance. An additional strain from 2016 (internal name ‘16.075’) was used in the Preference experiment. The cultures were established by flow cytometry-based single cell sorting of bee feces, then propagated at 27 °C, and preserved at −80 °C, as described previously^31^.

### Morphology Experiment

To determine morphological changes of parasites during selection for growth under high-phytochemical conditions, we used photographs taken during the aforementioned selection experiment that measured responses to eugenol-mediated selection^28^. These photographs compared the morphology of eugenol-exposed cell lines (grown in continuous presence of eugenol) and unexposed control cell lines (grown in continuous absence of eugenol) over the course of the 42 d exposure period, spanning approximately 100 generations. Two photographs of each line were taken under 400x magnification at the time of each cell transfer. From each photograph, 10 cells were haphazardly selected for measurement with ImageJ^32^, for a total of 100 measurements per eugenol treatment and time point. Cell cross-sectional area was recorded by tracing the cell perimeter, then calculating the area enclosed by the tracing; length was measured as distance along the major axis; width was measured perpendicular to the major axis at the widest point of the cell.

### Morphology: Analysis

All statistical analyses were conducted in R version 3.3^33^, and figures were made with the packages ggplot2^34^ and cowplot^35^. For analysis, each cell’s measurements were standardized relative to the mean of the control line at the corresponding time point. This controlled for any differences between treatments due to week-to-week differences in incubation and photographic conditions.

General linear mixed models were fit separately with either standardized area, length, or width as the response variable; eugenol exposure treatment and the eugenol x time of exposure interaction as predictor variables; and cell line within treatment as a random effect. Significance of individual terms was tested with chi-squared tests, which serve as alternatives to F-tests and do not require approximations to estimate denominator degrees of freedom^36^; chi-squared tests were conducted with the Anova function in the R package car^37^. Estimated group means for each time point were extracted with the lsmeans package^38^.

### Bee colonies

Experimental bee colonies were reared from *B. impatiens* queen bees collected near Normal, IL in April 2017. Workers from the same five colonies (internal numbers 17.003, 17.034, 17.049, 17.104, and 17.139) were used for both experiments. Colonies were maintained under red-light illumination in a climate-controlled room (26–29 °C) and provided with sugar water (1 g cane sugar:1 mL boiled tap water with 0.1% cream of tartar to promote sucrose hydrolysis) and honey bee-collected pollen (Brushy Mountain Bee Farms, Moravian Falls, NC). Colonies were confirmed to be free of common gut parasites, including *C. bombi*, by regular fecal screens of the original queen and subsequently produced workers.

To facilitate collection of age-controlled workers, all bees in the colony were marked on the dorsal thorax with white correction fluid at the start of the experiment. Thereafter, newly eclosed adult workers (identified by the absence of thoracic markings) were removed from the colony twice per week and acclimated in individual plastic enclosures for 2 d, with *ad libitum* access to sugar water and pollen, before inoculation and initiation of experimental diet treatments. Bees were, therefore, between 2 d and 6 d post-emergence at the time of inoculation.

### Infection Intensity Experiment

#### Experimental containers

Experimental bees were housed individually in 240 mL cylindrical polypropylene deli containers, with a drilled hole (8 mm diameter) to admit a feeder tube in the base. The feeder tube consisted of a 500 µL snap cap microcentrifuge tube with a 1.6 mm diameter hole drilled in the side at the 500 µL graduation. The tube was inserted tip-first into the hole. Tubes were filled with 500 µL 50% sugar water and exchanged daily.

#### *Crithidia* inoculations

Worker bees were moved to vented 20 mL vials and starved for 4 h prior to inoculation. Cell cultures, which had been thawed and cultured from 5 d prior, were diluted to 2× final density (2,000 cells µL^−1^) in growth medium, then mixed with an equal volume of sugar water to give a 1,000 cells µL^−1^ inoculum. Bees were inoculated with a 10 µL droplet of the inoculum (total dose: 10,000 cells bee^−1^). Bees were observed during inoculations; those that did not consume the droplet within 40 min were removed from the experiment. Post-inoculation, bees were moved to clean experimental containers and diet treatments were initiated.

#### Phytochemical diet treatments

Phytochemical diet treatments commenced immediately post-infection. The eugenol treatment consisted of 50 ppm (w/v) eugenol dissolved in sugar water. This concentration was chosen to match the concentration to which the evolved parasite lines had been chronically exposed during the *in vitro* experimental evolution of resistance experiment^28^. Ecologically, the 50 ppm concentration is higher than that observed in honeys (<1 ppm^39^), but similar to concentrations found in *Rosa x hybrida* stamens^40^, and well below concentrations found in leaves and whole flowers (e.g., 2400 ppm in *Ocimum selloi* flowers^41^). The 50 ppm eugenol concentration is also well below concentrations that increase mortality in *Apis mellifera* (8 d LD_50_ ~ 7800 ppm^42^), and 50 ppm eugenol in sugar water was attractive to free-flying bumble bees^43^. Eugenol concentrations in plant materials are summarized in^44^; effects on trypanosomatids are listed in^45^.

Eugenol treatments were prepared from a stock solution of 10 mg mL^−1^ pure eugenol in 95% ethanol. An equivalent amount of ethanol (0.5% volume) was added to the 0 ppm control solution to control for solvent effects. Bees were not fed pollen after inoculation, to avoid possible confounding effects of pollen phytochemicals on infection. Each bee was provided daily with 500 µL of the appropriate sucrose solution. Consumption was measured by weighing the tube before and after 24 h consumption periods. Consumption measurements were taken for 24–48 h and 120–144 h post-treatment initiation. Mass loss due to evaporation and handling was corrected by subtraction of the median mass loss of tubes in containers without bees; rates of mass loss in these control tubes did not differ across eugenol treatments (F_1,56_ = 0.33, *P* = 0.57).

#### Dissection and infection quantification

Bees were frozen at 7 d post-infection and kept at −80 °C until dissection. Bees were thawed and the intestinal tract was removed. The gut was cut at the junction of the mid- and hindgut, and the hindgut and rectum were frozen in 100 µL sterile Ringer’s solution until DNA extraction. Both forewings were also removed, and the marginal cell was measured as an index of bee size^46^, which was used as a covariate in statistical analyses.

Prior to extraction, gut samples were homogenized with a 100 mg sterile steel ball in 1.5 mL screw-cap tubes for 30 s at a speed of 5 m s^−1^ on a BeadRuptor (Omni International, Kennesaw, Georgia, USA). DNA was extracted from homogenized gut samples with the Qiagen (Hilden, Germany) DNeasy Blood and Tissue Kit following manufacturer’s instructions. DNA concentration and quality were checked by measuring absorbance at 260 and 280 nm on a microspectrophotometer.

Infection intensity was quantified as the amount of *C. bombi* DNA in the hindgut (measured in parasite cell equivalents), normalized to the amount of host actin DNA (measured as proportion of a reference extraction) to correct for differences in DNA extraction efficiency. Quantifications were made by quantitative polymerase chain reaction (qPCR) after^47^ on an ABI 7300 Real-time PCR machine. Each sample was run with two technical replicates. Absolute quantifications of *C. bombi* were made for each sample plate relative to an external standard curve of 8 dilutions of DNA extracted from *C. bombi* cell cultures (range: 1,563 to 100,000 cells per standard sample). Samples with a coefficient of variation >0.20 in the initial 2 technical replicates were rerun in duplicate. For rerun samples, results were averaged across all technical replicates after exclusion of anomalous values that differed from those of the other technical replicates by >2-fold. Infection intensity was normalized to content of *B. impatiens* actin in each sample, which was determined with a separate qPCR assay, again in duplicate for each sample, to control for differences in DNA extraction efficiency across samples. Primers for *B. impatiens* actin 5 C gene (NCBI Gene ID 100748723) developed by BMS (Forward: CAAACGCTCGCTCAAACG, Reverse: GTGTACGTGAATGGTCTTGCAC) were used with 10 min denaturation at 95 °C, followed by 40 amplification cycles of 15 s denaturation at 95 °C and 31 s simultaneous annealing and extension at 60 °C. Specificity was confirmed by melt-curve analysis. *Ct* values were converted by comparison with a standard curve of a dilution series of extracted DNA from 5 haphazardly selected bee guts from experimental bees: 40 µL of each of the 5 extracts were pooled, and 7 two-fold dilutions were prepared as templates. For our normalization, we took this mixture of bee guts to represent a typical extraction, and assigned the undiluted DNA extract a value of 1. Hence, the normalized infection intensity was computed as the ratio of *C. bombi* DNA relative to the standardized quantity of actin.

#### Infection Intensity Experiment: analysis

For infection intensity, normalized number of *C. bombi* cells per bee, rounded to the nearest whole number, was used as the response variable. Eugenol diet treatment, parasite selection regime (i.e., prior parasite exposure to eugenol), and their interaction were included as fixed effects, and forewing marginal cell length as a covariate. Date of inoculation was used as a random effect to account for the independently thawed and counted *C. bombi* aliquots used for the infection. Bee colony was initially included as a random effect, as the aim was to generalize the result across all bee colonies. However, because colonies exhibited such great variation in infection intensity, we did not deem it sensible to treat colony as a random effect, which would have obscured these clear colony-wise differences. Therefore, the model was re-fit with colony as a fixed effect to explicitly examine differences among colonies. The model used a negative binomial error distribution with zero inflation. The negative binomial is commonly used for non-negative count data that are over-dispersed relative to the Poisson distribution^48^; *Crithidia* infection intensities are often characterized by skewed distributions with long tails^46^. The zero-inflation parameter allows for the existence of two processes that can generate zero counts^49^, e.g., whether the infection initially became established, and whether the infection established but did not persist. Models were fitted in R package glmmTMB^50^. Significance of individual terms was tested with likelihood ratio chi-squared tests, conducted with the anova and drop1 functions, which compare the fits of models with and without the term under consideration. Estimated group means, confidence intervals, and pairwise comparisons were derived with the lsmeans package^38^.

For consumption, change in feeder tube mass was used as the response variable. Eugenol diet treatment, time since inoculation, the eugenol by time interaction, parasite adaptation, and wing size were included as fixed effects, and bee number nested within colony was included as a random effect to account for repeated measures. Residuals of a Gaussian model showed increased variance at higher fitted means. Therefore, we used a gamma distribution with a log link function^36^ to account for an increase of variance with mean. Parasite selection regime was initially included as a predictor, but removed from the final model because it did not explain significant variation in consumption (χ^2^ = 0.07, df = 1, *P* = 0.79).

### Preference Experiment

Worker bees, aged 0–4 d post-eclosion, were removed from the colony 2 d prior to parasite exposure. After isolation from the colony, worker bees were housed in clear plastic rectangular 340 mL deli containers (10 × 8 × 5 cm) with two 9.5 mm diameter holes drilled in the base 1 cm from each end. A microcentrifuge tube (0.5 mL, as used in the Infection Intensity Experiment) was placed in each hole. Bees were acclimated to the dishes for 2 d pre-infection. During the 2 d pre-infection acclimation period, both tubes contained 50% sugar water; bees were also provided with pollen paste.

After the 2 d acclimation period, infection-treatment bees were inoculated as in the Infection Intensity Experiment, but with 20,000 cells per bee (10,000 cells each of the ancestral Infection Intensity Experiment cell line IL13.2 and cell line 16.075, both naïve to eugenol). Control-infection bees were sham-inoculated with sugar water mixed with growth medium, but without parasites. Based on compliance with inoculation observed in the Infection Intensity Experiment, twice as many bees were assigned to the infection treatment as to the sham-inoculation treatment; we expected approximately half of the infection-treatment bees to refuse the inoculum. However, unexpectedly high compliance during inoculation resulted in a larger sample size in the infection treatment (n = 44 bees) than in the sham-inoculation treatment (n = 25 bees).

After inoculation, bees were returned to their individual containers. One feeder tube was filled with 50 ppm eugenol-containing sugar water, and the other with 0 ppm eugenol sugar water (the same concentrations used in the Infection Intensity Experiment). The location of the eugenol tube was selected randomly (by coin flip). Thereafter, the eugenol tube was placed in same hole each day to allow bees to associate eugenol with its location. Tubes were replaced daily with fresh solutions, and consumption was measured at 24 h intervals beginning at 24 h post-infection and continuing through 6 d post-infection, as outlined in the Infection Intensity Experiment. Wing measurements were taken for use as a covariate.

### Preference: analysis

To analyze consumption, change in feeder tube mass was used as the response variable. Eugenol diet treatment, infection treatment, and wing size were included as fixed effects. Bee number nested within colony were included as random effects to account for repeated measures, and trial date was included as an additional random effect to account for non-independence of consumption from each of the two solutions during any given 24 h feeding interval. The model used a gamma distribution with a log link function to account for higher variance at higher means. Time since inoculation was initially included as a predictor, but removed from the final model because it did not explain significant variation in consumption (χ^2^ = 0.31, df = 1, *P* = 0.57).

### Data availability statement

All data generated or analyzed during this study are included in this published article and its Supplementary Information files (see Supplementary Data S1).

## Results

### Morphology

**Figure 1:**
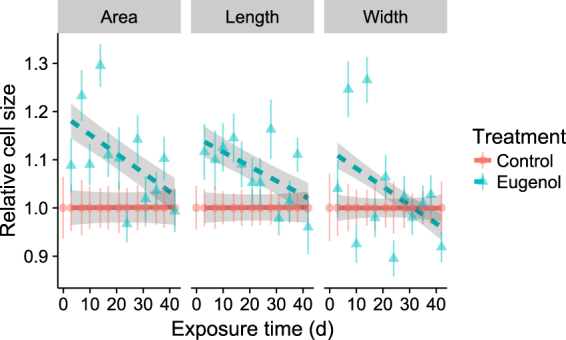
Effects of chronic 50 ppm eugenol treatment and time (cumulative duration of chronic exposure) on *C. bombi* cell morphology *in vitro*. At each time point, we measured area, length, and width of 20 cells for each of 5 cell lines per treatment. Size measurements were standardized relative to the mean size of cells in the control cell lines at the corresponding time point. Lines and shaded bands represent model means and 95% confidence intervals. Points and error bars represent raw means and 95% confidence intervals.

Eugenol had strong initial effects on cell size that significantly diminished over multiple generations of chronic exposure. During the initial weeks of the selection regime, cells of the exposed lines (grown in continuous presence of eugenol) exhibited up to 30% increase in cross-sectional area relative to cells of the control lines (grown in continuous absence of eugenol), which reflected increases in both length and width (Fig. 1, Table 1). We observed that the cells would often fold or curl along the major axis; this resulting in a swollen and rounded appearance. However, all three morphology metrics were similar to the control by the completion of the 42 d selection regime (Fig. 1), as reflected by the significant interaction between the effects of eugenol exposure and time (Table 1).

**Table 1:** Effects of chronic 50 ppm eugenol treatment and time (cumulative duration of chronic exposure) on *C. bombi* cell morphology *in vitro*.

**A. Area**	**χ2**	**df**	**P**
Eugenol	52.801	1	<0.001
Eugenol × Time	48.422	2	<0.001
**B. Length**	**χ2**	**df**	**P**
Eugenol	38.136	1	<0.001
Eugenol × Time	32.203	2	<0.001
**C. Width**	**χ2**	**df**	**P**
Eugenol	31.829	1	<0.001
Eugenol × Time	47.327	2	<0.001

At each time point, we measured (A) area, (B) length, and (C) width of 20 cells for each of 5 cell lines per treatment.

### Infection

**Figure 2:**
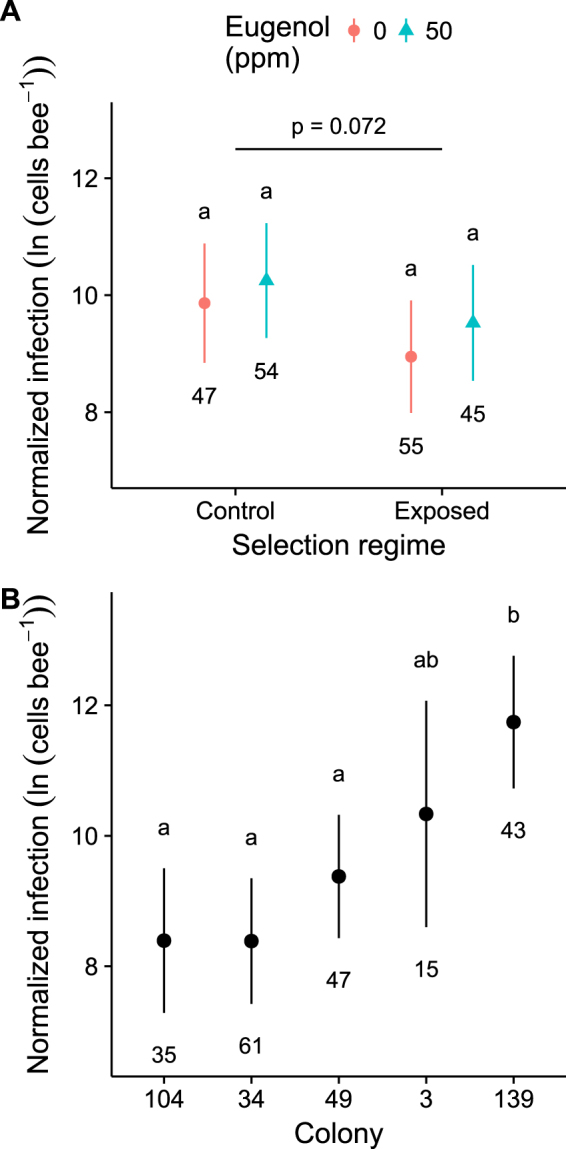
Effects of (**A**) eugenol consumption and parasite selection regime and (**B**) bee colony on infection intensity in *B. impatiens*. Bees from each of 5 colonies were fed 0 or 50 ppm eugenol after infection with *C. bombi* lines (control or selected for eugenol resistance). Points and error bars represent model means and 95% confidence intervals. Numbers indicate sample size. Different lower case letters represent significant differences in *post hoc* pairwise comparisons for effects of eugenol diet treatment given parasite selection regime in (**A**) or across colonies in (**B**). Horizontal line with *P-*value indicates pairwise comparison between eugenol-selected and control lines.

We found that neither eugenol treatment nor prior parasite adaptation to eugenol significantly affected infection intensity (Table 2). There was a pattern of 55% lower normalized infection intensity in bees infected with the lines selected for eugenol resistance (covariate-adjusted log mean: 9.24 ± 0.38 SE for eugenol-selected lines vs. 10.05 ± 0.38 SE for control lines). However, this effect was not statistically significant across the five experimental colonies (*post hoc* pairwise comparison: *Z* = 1.80, *P* = 0.072, Fig. 2A, Table 2).

**Table 2:** Predictors of infection intensity in *B. impatiens* fed 0 or 50 ppm eugenol after infection with *C. bombi* lines (control or selected for eugenol resistance).

Term	χ2	df	P
Eugenol	1.189	1	0.276
Selection regime	3.120	1	0.077
Eugenol × Selection regime	0.040	1	0.841
Colony	33.204	4	<0.001
Wing size	6.598	1	0.010

Wing size refers to the length of the forewing marginal cell.

Infection was strongly affected by colony (Fig. 2B, Table 2), consistent with prior work on genotype-genotype interactions in the *Bombus-Crithidia* system^23^. Normalized mean infection intensity in colony 139 was at least 0.99 log-units (2.69-fold) higher than infection in any of the other 4 colonies (Fig. 2B). Differences in median normalized infection intensity were even more striking (median 9,805 cells bee^−1^ in Colony 139 vs. 7 to 83 cells bee^−1^ in the other four colonies). There were no significant effects of either eugenol, parasite selection, or their interaction in a follow-up model that considered only the 43 bees in this more heavily infected colony (*P* > 0.29 for all).

Infection intensity was negatively correlated with wing size (β = −3.07 ± 1.20 SE, *Z* = 2.56, Table 2), indicative of higher parasite resistance in larger bees. This is consistent with previous results in which larger bees had lighter infections^16^. Levels of the reference gene (*B. impatiens* actin 5 C) did not differ across eugenol diet or parasite selection treatments, or their interaction (*P* > 0.22 for all). Levels of actin 5C were positively but weakly correlated with wing size (β = 0.33 ± 0.12 SE*, T* = 2.78, *P* = 0.006, r^2^ = 0.032).

### Consumption

**Figure 3:**
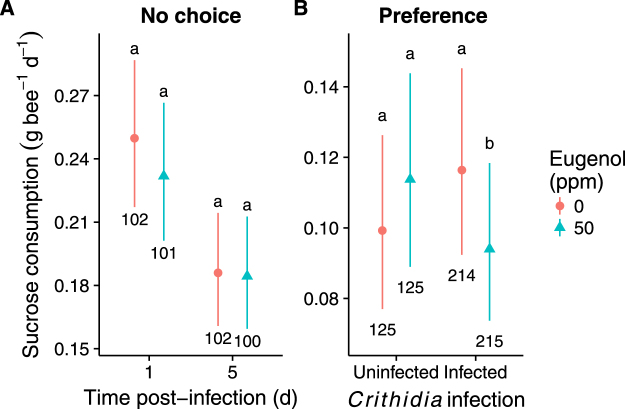
Effects of eugenol treatment on sugar water consumption by *B. impatiens* under (**A**) no-choice conditions in the Infection Intensity Experiment and (**B**) choice conditions in the Preference Experiment. Points and error bars represent model means and 95% confidence intervals. Numbers indicate sample size. Different lower case letters represent significant differences in *post hoc* pairwise comparisons for effects of eugenol diet treatment given time post-inoculation in (**A**) or infection treatment in (**B**).

Under the no-choice conditions of the Infection Intensity Experiment, neither parasite selection treatment (χ^2^ = 0.07, df = 1, *P* = 0.79; removed from final model) nor eugenol treatment significantly affected sucrose consumption (Table 2A, Fig. 3A). Consumption was significantly (23%) lower at 5 d vs 1 d post-infection, but there was no significant eugenol by time interaction (Table 3). Consumption was positively correlated with wing size (β = 0.20 ± 0.080 SE, *Z* = 2.45), indicating that larger bees consumed more (Table 3).

**Table 3:** Effects of dietary eugenol treatment (0 or 50 ppm) on sugar water consumption by *B. impatiens* under (A) no-choice conditions in the Infection Intensity Experiment and (B) choice conditions in the Preference Experiment.

A. No choice	χ2	df	P
Eugenol	1.174	1	0.279
Time	30.758	1	<0.001
Eugenol × time	1.577	1	0.209
Wing size	5.908	1	0.015
**B. Preference**	**χ2**	**df**	**P**
Eugenol	3.812	1	0.051
Infection	0.088	1	0.767
Eugenol × Infection	15.228	1	<0.001
Wing size	18.118	1	<0.001

Wing size refers to the length of the forewing marginal cell. Parasite selection regime in the no-choice experiment and time since inoculation in the preference experiment were not significant predictors of sugar water consumption. These terms were therefore removed from the final model.

Under the choice conditions of the Preference Experiment, *Crithidia* infection treatment did affect preference for eugenol versus eugenol-free sugar water, as indicated by the significant eugenol x infection interaction (Table 3B), but results were contrary to our expectation that infected bees would prefer eugenol. Instead, uninfected bees displayed a non-significant preference (15% higher consumption) for the eugenol-containing solution (*Z* = 1.92, *P* = 0.055); however, infected bees had a significant preference (24% higher consumption) for the eugenol-free sugar water (*Z* = 3.95, *P* < 0.001, Fig. 3B). There were no main effects of infection (Table 3B) or time since inoculation (χ^2^ = 0.31, df = 1, *P* = 0.57; dropped from final model) on consumption. As in the Infection Intensity Experiment, consumption was positively correlated with wing size (β = 0.43 ± 0.094 SE, *Z* = 4.63, Table 3B).

## Discussion

Our *in vitro* experiments indicated strong effects of eugenol on cell morphology that subsided over 6 weeks’ continued culture under chronic exposure. These results mirror previously reported decreased susceptibility to eugenol of these same lines^28^. However, eugenol consumption by bumble bees had no effect on *in vivo* infection level, and *in vitro* eugenol adaptation was neutral, or even potentially costly, for infectivity, regardless of the eugenol content of bee diets. Moreover, parasite infection shifted bee diet preferences away from eugenol-containing sucrose solutions. We discuss the implications of these results for plant-pollinator-parasite ecology and evolution.

Eugenol exposure inhibited *in vitro* growth^28^ and, as shown in this study, led to cellular enlargement of *C. bombi*. These morphological changes are consistent with the effect of eugenol-rich plant extracts on *Trypanosoma cruzi*, which became swollen and rounded after 24 h incubation in clove oil (86% eugenol^51^). However, these increases in *C. bombi* cell size disappeared almost completely after 6 weeks of chronic exposure (Fig. 1). The observed normalization in cell morphology could be a consequence of the evolution of resistance, as demonstrated by previous growth measures^28^, but acclimation and transgenerational effects cannot be discounted in this particular case. To distinguish these possibilities, it would be necessary to relax selection on eugenol-exposed lines by transferring them to eugenol-free medium, and then to compare morphology of eugenol-exposed and eugenol-naïve cell lines after a brief re-exposure to eugenol. However, the reduction in the effects of eugenol on morphology were accompanied by increased 50% inhibitory concentrations and diminished effects of the fixed 50 ppm exposure concentration on growth^28^. Notably, the 50% inhibitory concentrations were measured after a 48 h relaxation of eugenol-mediated selection^28^, and therefore constitute the strongest evidence of the evolution of eugenol resistance.

Despite its strong effects on *in vitro* growth and morphology, eugenol did not alter infection when added to the diets of bumble bees at the 50 ppm concentration used in the exposure treatment. This concentration had no effect on infection with either the control or eugenol-selected cell lines, despite the fact that 50 ppm eugenol inhibited *in vitro* growth of un-adapted cell lines by >50%^28^. Although not measured here, we speculate that the lack of effect of oral eugenol on infection reflects intestinal absorption and metabolism of eugenol by bumble bees, which likely led to relatively low phytochemical concentrations in the distal gut. *Crithidia* bombi and its honey bee-infecting relative *Lotmaria passim* are found mainly in the hindgut^52,53^. Before reaching the hindgut, oral ingesta must pass through the midgut, which contains absorptive surfaces and detoxification enzymes. Eugenol, like many other plant volatiles, is a relatively nonpolar compound that easily crosses membranes^54,55^, which likely facilitated diffusion out of the crop and midgut lumen. Phytochemicals may also be chemically modified by intestinal cytochrome p450 enzymes; for example, little nicotine reached the hindgut in honey bees fed 50 ppm nicotine in sugar water^56^. Therefore, the amount of eugenol in the hindgut, where *C. bombi* establishes, may have been too low to affect growth of either susceptible or resistant cell lines. These absorptive and metabolic processes may likewise explain why thymol ingestion did not affect *C. bombi* infection^19^ when consumed at doses high enough to inhibit parasite growth *in vitro*^45^, although empirical measurements of hindgut phytochemical concentrations are necessary to test this hypothesis.

The lack of concordance between *in vitro* and *in vivo* effects may be illustrated by analogy with the effects of sugar. Addition of 20% sugar to growth medium completely inhibited growth of *C. bombi in vitro*^57^. However, alteration of dietary sugar concentration across several orders of magnitude had no consistent effects on *Crithidia* establishment in either *B*. *impatiens* or *B. terrestris*^21,22^. Efficient hydrolysis and absorption of sugar in the proximal intestine of bees and other nectar-feeding insects may explain this discrepancy^58^. Because relatively little sugar reaches the hindgut, hindgut parasites are unlikely to experience sugar-mediated osmotic pressure, and dietary concentrations likely have minimal direct effects on hindgut parasite growth. Similarly, our results indicate that eugenol consumption does not affect *C. bombi* persistence and reproduction within bumble bee hosts.

Although eugenol may have little effect on within-host *C. bombi* growth, it may still have ecologically relevant effects on parasite transmission that occurs via shared flowers^59,60^. Between-host transmission can exert selection for different *C. bombi* genotypes^61^, and can involve exposure to higher phytochemical concentrations than those found in either nectar or honey^41,44,45^. Future experiments should evaluate the direct effects of eugenol and other floral volatiles on horizontal transmission of *Crithidia*, and whether these direct effects are mitigated in phytochemical-resistant cell lines.

We found suggestive but statistically non-significant effects of selection for phytochemical resistance on within-host infectivity. However, it was clear that evolved resistance to the phytochemical eugenol offered no benefit in terms of within-host infectivity, irrespective of the presence of the phytochemical in the host’s diet. By comparison, studies of drug resistance in human-parasitic protozoa have shown costs (reviewed in ref.^27^), benefits^62,63^, and no effects^64^ of chemical resistance on within-host infectivity.

In natural populations, factors other than selection for phytochemical resistance may have stronger effects on parasite evolution than do phytochemicals, and may counteract the evolution of resistance in nature^65^. In our study, we found that bee colony—corresponding to host genotype—was a stronger predictor of infection success than either dietary eugenol or prior selection for eugenol resistance. In all but one colony, infection success was relatively low. This colony-dependent variation is consistent with previous work that showed strong genotype-by-genotype interactions in host immune responses and infection intensity; some host colonies appear susceptible to almost any *C. bombi* strain, whereas others are largely resistant^66^. Hence, negative frequency-dependent selection on bee and parasite genotypes may exert stronger and more sustained effects on parasite evolution than do phytochemicals, which can vary spatially and temporally across habitats. In addition, queen migration and parasite genetic drift or unrelated selection during queen hibernation may counteract adaptation to local phytochemical environments. Therefore, the observed varied and submaximal eugenol resistance in *C. bombi*^28,45^ may reflect an apparent absence of selection for eugenol resistance during within-host replication, possible costs to infectivity, and the presence of other evolutionary influences that include host genotypic composition, migration, and genetic drift.

We found that infection altered relative dietary preferences to favor eugenol-free over eugenol-containing sugar water. This contrasts with previous studies in which insects favored diets higher in potentially toxic plants or phytochemicals when infected^6,9,10,15,26^. One difference between our study and those in which infected bumble bees were attracted to high-phytochemical flowers is that our study used caged bees and measured consumption over a longer period, whereas other studies used proportional visitation rate^15^ and time spent per flower^26^ as response variables. Bees can readily perceive eugenol, which is attractive at up to 50 ppm in sugar water^43^ and stimulates pollen collection^67^. However, chronic consumption of eugenol in caged bees introduces the potential for results to be affected by toxicity and malaise as well as initial preference, although we found no time by treatment interaction. In honey bees, infection decreased tolerance of dietary nicotine^68^. It is possible that infected bees faced trade-offs between immunity and detoxification^69^ that caused them to favor low-eugenol diets when infected. Given that eugenol consumption did not decrease infection, this may be a choice that is adaptive for the host. Alternatively, this aversion could represent parasite manipulation^1^ to repel bees from high-eugenol flowers that could curtail horizontal transmission. Experiments under different nutritional and behavioral contexts would help to clarify the extent to which floral eugenol influences bumble bee foraging behavior and pollination services, and whether changes in pollinator infection can exert pollinator-mediated selection on floral phytochemical composition of nectar, pollen, and other tissues. Our results suggest the hypothesis that infection could favor lower-eugenol plant individuals and taxa.

In summary, eugenol caused morphological changes concurrent with growth inhibition^28^ that could alter viability during horizontal transmission at eugenol-rich flowers. These changes were mitigated by adaptation, which has the potential to benefit horizontal transmission in high-eugenol floral environments. However, eugenol consumption by bees did not alter infection, indicating that there is likely little selective pressure for eugenol resistance during within-host growth. Moreover, selection for growth under eugenol exposure resulted in suggestive but non-significant changes in infectivity, in line with a cost of evolved phytochemical resistance. Together, the lack of benefits, possible costs, and presence of other strong selective forces during within-host replication may contribute to varied and submaximal eugenol resistance in *C. bombi* populations. The effects of infection on host phytochemical preference suggest future research on how infection alters pollination services and selection on floral traits.

## Electronic supplementary material

Supplementary Data S1
